# Notes and News

**Published:** 1903-10-17

**Authors:** 


### Notes and News.

The King has signified his pleasure that the title of "Royal" shall be continued in connection with the name of the Royal Hospital for Children and Women, Waterloo Bridge Road. The hospital, which was founded by the Duke of Kent and Sussex, is the oldest of its kind in the kingdom.

The report on the result of anti-typhoid inocculation both in India and South Africa shows that, if not infallible in effect, it undoubtedly diminishes the danger of infection, and the severity of the nature of the disease where it fails to secure immunity.

The usual Harvest Festival Service took place in the chapel of the National Hospital for the Paralysed and Epileptic last week, when the Rev. E. C. Bedford, the vicar of St. George the Martyr, Bloomsbury, gave an address. The chapel had been very tastefully decorated for the occasion by the night sister and night nurses (male and female), a large proportion of the floral adornments having been supplied from the gardens of the convalescent home at East Finchley. There was a full attendance of patients, nurses, and friends of the hospital, among whom were the chairman (Mr. J. Danvers Power) and the vice-chairman (Sir John R. Paget, Bart, K.C).

Dr. Collingridge, in observations on the suppression of the spitting habit, points out that the crusade should commence in school life by means of the teachers. Possibly a few stray seeds of wisdom thus sown may bear fruit, but with the home example at work in direct opposition to the teaching abroad, the result at first can but be slow. The small boy naturally admires and imitates all the characteristics, good or bad, which distinguish the bearing of the grown-up man from the youthful population. The mind of the parent must be prepared also, or stormy scenes will ensue when the pupil of the new doctrine returns home to inquire why his father resorts to a habit described by the teacher as "filthy."

The epidemic of plague in Mauritius shows no abatement, but the percentage of mortality is not quite so great.

The appointment has been made of Dr. C. J. Martin, Professor of Physiology at Melbourne, to the Directorship of the Lister Institute of Preventive Medicine.

Scarlet fever is very prevalent in Edinburgh, and typhoid and diphtheria cases are more numerous than usual. Although there is a great deal of illness generally in the city, the death-rate is lower than usual.

We fully endorse the views of the *British Medical Journal* that fishmongers should name the place of supply from which the oysters that they sell are drawn. The comment of the Fishmongers' Company to this proposal which emanated from Dr. Collingridge should not be regarded as satisfactory. They plead that fishmongers who followed the proposal would be unduly advertised. Seeing that every fishmonger could place himself upon the list, it is only right that the public should have the opportunity to patronise tradesmen who prove themselves as endeavouring that their wares should be above suspicion.

An interesting meeting of the African Trade Section Liverpool Chamber of Commerce was held recently when a communication was reported from the Cape Coast Chamber of Commerce calling attention to the greatly improved sanitary condition of the inhabitants of Cape Coast, owing to the sanitary measures that have been taken for some time past. One marked feature was the diminution, amounting almost to total disappearance, of small-pox. In connection with West Africa generally, the Liverpool committee approached the Government with a recommendation that a complete system of sanitary inspection should be adopted on the Indian model, including the publication of statistical reports. Mr. Chamberlain's reply did not favour the adoption of so expensive a scheme, as he questioned its utility at the present stage of development in West Africa.

A serious blow is aimed at cleanliness through the medium of a plentiful use of soap by one who signs himself as a Colonist in Cape Colony when writing to the press on the subject. He maintains that humanity is given a natural oil to protect its skin, which it ruthlessly removes by the use of soap, thus exposing itself to the inroads of rheumatism, and all manner of diseases. He considers that the deterioration of the race is most marked amongst the genteel community. The theories which our Colonist propounds are very amusing, but it is to be hoped for his own sake that he will not be called upon to supply chapter and verse for his very rash assertions. We can at any rate assure him that the poorer section of our population is not at the present time in any danger of deterioration if that deterioration is dependent on the daily subjection of their bodies to a course of soap and water. The genuine alarm with which the hospital patient regards the infliction of a bath is enough testimony to prove this.

